# Cytokine expression and cytokine‐mediated cell–cell communication during skeletal muscle regeneration revealed by integrative analysis of single‐cell RNA sequencing data

**DOI:** 10.1002/ccs3.12055

**Published:** 2024-12-04

**Authors:** Pallob Barai, Jie Chen

**Affiliations:** ^1^ Department of Cell and Developmental Biology University of Illinois at Urbana‐Champaign Urbana Illinois USA; ^2^ Department of Biomedical and Translational Sciences Carle Illinois College of Medicine Urbana Illinois USA

**Keywords:** cell–cell communication, cytokines, muscle stem cells, scRNA‐seq, skeletal muscle regeneration

## Abstract

Skeletal muscles undergo self‐repair upon injury, owing to the resident muscle stem cells and their extensive communication with the microenvironment of injured muscles. Cytokines play a critical role in orchestrating intercell communication to ensure successful regeneration. Immune cells as well as other types of cells in the injury site, including muscle stem cells, are known to secret cytokines. However, the extent to which various cell types express distinct cytokines and how the secreted cytokines are involved in intercell communication during regeneration are largely unknown. Here we integrated 15 publicly available single‐cell RNA‐sequencing (scRNA‐seq) datasets of mouse skeletal muscles at early regeneration timepoints (0, 2, 5, and 7 days after injury). The resulting dataset was analyzed for the expression of 393 annotated mouse cytokines. We found widespread and dynamic cytokine expression by all cell types in the regenerating muscle. Interrogating the integrated dataset using CellChat revealed extensive, bidirectional cell–cell communications during regeneration. Our findings provide a comprehensive view of cytokine signaling in the regenerating muscle, which can guide future studies of ligand‐receptor signaling and cell–cell interaction to achieve new mechanistic insights into the regulation of muscle regeneration.

## Graphical abstract

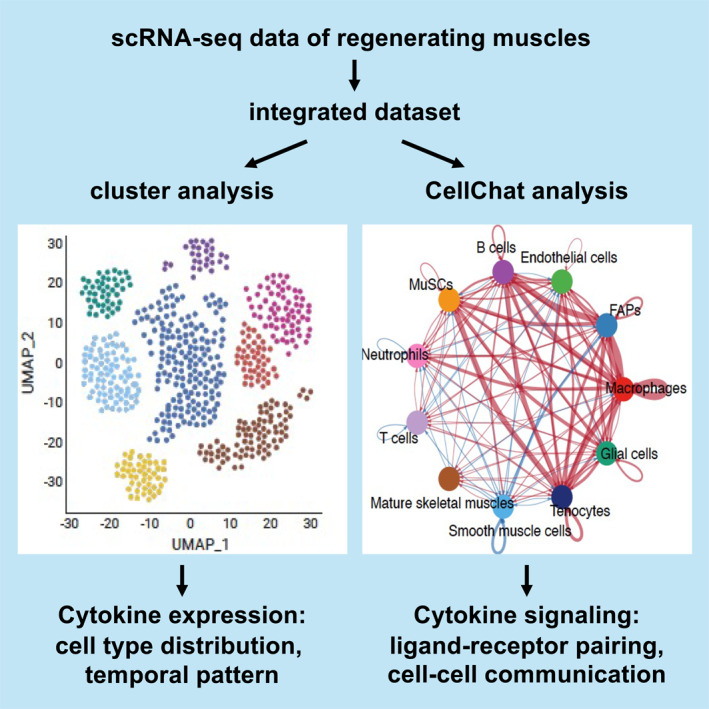

## 1 INTRODUCTION

Skeletal muscles possess the inherent capability to undergo self‐repair upon injury, owing to the presence of resident muscle stem cells (MuSCs). A heterogeneous population of interacting immune cells, fibro‐adipogenic progenitors (FAPs), endothelial cells, smooth muscle cells, and myogenic cells contributes to the regulation and maintenance of MuSC quiescence, activation, proliferation, and differentiation.^1^, ^2^, ^3^, ^4^, ^5^, ^6^ MuSCs are a rare type of cells, constituting less than 2%–7% of all cells in homeostatic skeletal muscles. During injury‐induced regeneration, a subset of activated Pax7^+^ MuSCs return to quiescence, and the rest further proliferate, generating Myod1^+^ myoblasts, which undergo differentiation to result in Myog^+^ myocytes and fusion to form new myofibers or repair injured myofibers.^7^, ^8^

Although MuSCs are indispensable in muscle regeneration, other cell types are also necessary for proper regeneration and repair of damaged muscles. Macrophages play essential roles by clearing damaged tissues and supporting MuSC proliferation and differentiation; systemic removal of macrophages impairs muscle regeneration.^9^, ^10^, ^11^ FAPs are a population of muscle‐specific mesenchymal cells that remain in quiescent state in the uninjured muscle.^3^, ^6^ Depletion of FAPs in uninjured muscles induces muscle atrophy and reduction in MuSCs numbers, suggesting a crucial role of FAPs in maintaining muscle homeostasis.^12^ In the injured muscle, depletion of FAPs results in decreased expansion of MuSCs and CD45^+^ hematopoietic cells and defect in regeneration.^12^ Endothelial cells are necessary for vascularizing the regenerating tissues through angiogenesis, and they also regulate myogenic cell differentiation and quiescence.^13^ Recently, tenocytes and smooth muscle mesenchymal cells have been identified in the adult muscle.^14^ It is suggested that tenocytes support muscle regeneration by collaborating with FAPs to remodel ECM.^13^ While it is evident that many types of cells contribute to muscle regeneration, how these cell types communicate with each other to regulate regeneration remains poorly understood.

In response to injury, inflammatory signals such as cytokines and chemokines (collectively referred to as cytokines herein) activate and recruit platelets, macrophages, and neutrophils to the injury site, which are required for the clearance of damaged tissues and the initiation of regeneration.^9^, ^10^, ^11^, ^15^, ^16^ Tissue resident macrophage‐secreted cytokines, such as TNF‐α, IFN‐γ, CXCL1, and CCL2, promote the recruitment of neutrophils and pro‐inflammatory macrophages (M1 macrophage) to clear the debris and to subsequently initiate regeneration.^10^ Although the importance of immune cell‐derived cytokines in muscle regeneration has long been established, it has become increasingly evident that other types of cells in the regenerating muscle, including MuSCs, also secrete cytokines. Many of those cytokines have been shown to regulate various stages of myoblast differentiation in vitro and muscle regeneration in vivo.^17^ However, a comprehensive analysis of cytokine expression by all cell types in the regenerating muscle has not been reported.

Single‐cell RNA sequencing (scRNA‐seq) has revolutionized our understanding of the transcriptomes of injured muscles by revealing a remarkable diversity in gene expression among different cell types and identifying new cell populations involved in the process of regeneration (e.g., 18, 19, 20, 21, 22). With these publicly available scRNA‐seq databases, high‐resolution analysis of cytokine expression by various cell populations in regenerating muscle is now possible. In the current study we integrated several scRNA‐seq datasets collected at various timepoints of injury‐induced regeneration of mouse muscles by leveraging recent improvements in batch‐correction algorithms.^23^ The integrated data were then analyzed for the expression of 393 known mouse cytokines during regeneration. We further extended our analysis to identify cytokine‐mediated cell–cell communication networks. Our results reveal widespread and temporally dynamic expression of cytokines in the regenerating muscle niche and highlight a cytokine‐mediated communication landscape during muscle regeneration.

## 2 METHODS

### 2.1 Preprocessing, quality control, and batch correction of scRNA‐seq data

Raw sequencing reads were downloaded from the Sequence Read Archive (SRA) databases. The Cell Ranger (v3.1.0) pipeline^24^ was used to align the datasets to the mouse genome (GRCm39) and create raw gene expression matrix. The raw gene expression matrix was filtered, normalized, and clustered using Seurat (v4.4.0).^23^ Cells containing fewer than 500 features or more than 10% unique transcripts from mitochondrial genes were removed. After filtering, standard Seurat workflow (normalization, finding variable features, RunPCA, finding neighbors, finding clusters, and RunUMAP) was performed on each dataset. SCTransform v2^25^, ^26^ was used to normalize the datasets. RunPCA function in Seurat was used to decrease the linear dimensionality. After preprocessing the data, the datasets were merged and unintegrated analysis was performed to identify any batch effects. After confirming minimal batch effects, the Seurat data integration method was used to correct batch effects and perform comparative analysis across different experimental conditions.^23^

### 2.2 Cell type annotation

Initially, the raw clustering of the integrated dataset resulted in 31 different clusters (cluster resolution 0.8). Expression patterns of canonical marker genes were used to define the various cell clusters. Clusters that had similar marker expression patterns were merged, which resulted in a final 11 distinct cell clusters. A list of markers used to identify each cluster can be found in Table S1.

### 2.3 Cell–cell communication analysis with CellChat

Cellular communication network analysis and visualization were performed using CellChat (version 2.1.2) (https://github.com/jinworks/CellChat) on the integrated datasets. To identify cytokine‐mediated communications, we subset the database only for secreted factor signaling interactions. To predict cell–cell communication, CellChat identifies overexpressed ligands or receptor in one cell type and infer the ligand–receptor interactions if either ligands or receptors are overexpressed in other cell types. The “identifyOverExpressedGenes” function was used to identify genes that were overexpressed and then the gene expression data were projected onto protein–protein interaction network. To infer communication networks, we used “computeCommunProb” with the parameter “type = triMean” and filtered out cell–cell communications that were present in less than 10 cells using the “filterCommunication” function. We used “aggregateNet” to quantify the total number of communications in the integrated datasets. To reveal changes in sending or receiving signals for each cell type during regeneration, “netAnalysis_signalingRole_scatter” was used. The “computeCommunProbPathway” function was used to sum the communication probabilities of all ligand–receptors interactions for each signaling pathway. To compare the overall information flow between different timepoints, we used “rankNet” with “do.stat = TRUE”, and the paired Wilcoxon test was run to identify significant differences of the information flow between two timepoints. To identify upregulated ligand–receptor pairs during regeneration we compared each regenerating timepoint to 0 dpi using “identifyOverExpressedGenes” with the following parameters: “thresh.pc = 0.1”, “thresh.fc = 0.05”, and “thresh.p = 0.05”. To visualize upregulated ligand–receptor pairs, “netVisual_bubble” was used with “source.use” and “target.use” to specify the targets and sources of signals.

### 2.4 Data availability

All datasets analyzed in this study are published.^18^, ^19^, ^20^ GEO accession information for the datasets can be found in Table S2. Codes and integrated datasets are available on reasonable request.

## 3 RESULTS

### 3.1 Integration of scRNA‐seq datasets provides a high‐resolution view of regenerating muscle transcriptomics

**FIGURE 1:**
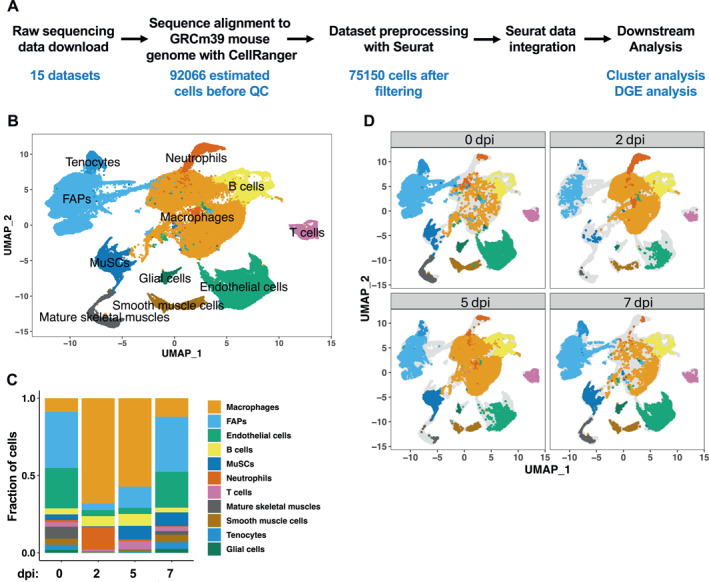
Integration of 15 scRNA‐seq datasets at various days post injury (dpi) of TA muscles and analysis of cell types during regeneration. (A) A schematic of scRNA‐seq data integration and downstream analysis. (B) Single‐cell atlas of 75,150 cells assembled from all the timepoints. Data are presented in UMAP to visualize the transcriptional differences between different cell clusters. (C) Cell type composition throughout the early phase of regeneration. (D) Single‐cell atlases at 0, 2, 5, and 7 dpi.

To comprehensively evaluate the transcriptomic profiles of mouse skeletal muscles at the single‐cell level during homeostasis and regeneration, we curated 15 publicly available scRNA‐seq datasets generated from uninjured and injured mouse tibialis anterior (TA) muscles at various timepoints^18^, ^19^, ^20^ (Table S2). The raw data were aligned to the reference mouse genome with Cell Ranger^24^ and processed with Seurat^23^ to construct uniform manifold approximations and projections (UMAP) ^27^ for all datasets. Unintegrated clustering analysis revealed that each of the datasets showed similar clustering behavior regardless of their sources and injury methods (Figure S1), suggesting that there was little batch effect. The datasets were then aggregated with Cell Ranger, generating four datasets for 0, 2, 5, and 7 days post injury (dpi), respectively. An estimated total of 92,066 cells were processed, and after filtering for high‐pass feature counts 75,150 cells were integrated using Seurat (Figure 1A). Integration and unsupervised clustering by UMAP returned 11 distinct clusters, with transcriptomic profiles identifying with macrophages, FAPs, MuSCs, endothelial cells, smooth muscle cells, mature skeletal muscles, glial cells, neutrophils, tenocytes, B cells, and T cells (Figure 1B). A heatmap visualization of the top 10 most variably expressed genes in each cluster revealed a distinct transcriptional program and expression of known lineage‐specific transcripts (Figure S2A). Macrophages constituted ∼37% of the total population based on the expression of the canonical macrophage markers Itgam, Csfr1 and Adgre1 (Figures 1C and S2B). FAPs expressing Pdgfra and Ly6a were the second most abundant cell population at ∼22%, whereas ∼5.7% of the total population (4335 cells) were assigned to MuSCs expressing the myogenic transcription factors Pax7 and Myod1 (Figures 1C and S2B).

To determine how the cell populations may change during the process of regeneration, we used the split plot function in Seurat to visualize single cells at their respective timepoints (Figure 1D). Uninjured muscles (0 dpi) were mostly composed of FAPs, endothelial cells, macrophages, and mature skeletal muscles. As expected, a transient increase in multiple immune cell populations after injury was observed: at 2 dpi and 5 dpi, immune cells comprised the largest portion of the total population, whereas at 7 dpi, their numbers returned to those of 0 dpi (Figures 1C and 1D). On the other hand, the numbers of FAPs and endothelial cells decreased drastically at 2 dpi and returned to their normal levels at 7 dpi (Figures 1C and 1D). In the uninjured state, ∼3.8% of the total cells were MuSCs, but at 2 dpi, very few MuSCs were detected (0.6%). The rarity of MuSCs at 2 dpi might be due to technical difficulties in their recovery from tissues with a high abundance of immune cells as previously suggested.^18^, ^20^ A drastic increase in the MuSC population was observed at 5 dpi and 7 dpi (Figures 1C and 1D) as expected.

To further validate the integrated datasets, we analyzed the temporal dynamics of gene expression by macrophages, FAPs, and MuSCs. As shown in Figure S3A, macrophages presented a transcriptional profile reflecting a pro‐inflammation‐to‐anti‐inflammation switch from early to later phase of regeneration as reported^18^, ^20^: expression of Chil3, Ptgs2 and Cxcl3 at 2 dpi, and increased expression of Apoe, Ms4a7, Lsp1, and H2‐Aa at 5 and 7 dpi. The temporal profile of gene expression in FAPs (Figure S3B) was consistent with a transition from homeostasis at 0 dpi (expression of ECM genes Dcn, Gsn, and Dpp4) to activation and remodeling as marked by the upregulation of Bgn, Mest, Postn, and Col1a2,^18^, ^20^ and returning to the homeostatic state at 7 dpi. Finally, the expression profile of MuSCs followed the anticipated progression from quiescence to activation, proliferation, and differentiation (Figure S3C). Taken together, these results fully validate our methods of data integration.

### 3.2 Cytokine expression across all cell types during muscle regeneration

**FIGURE 2:**
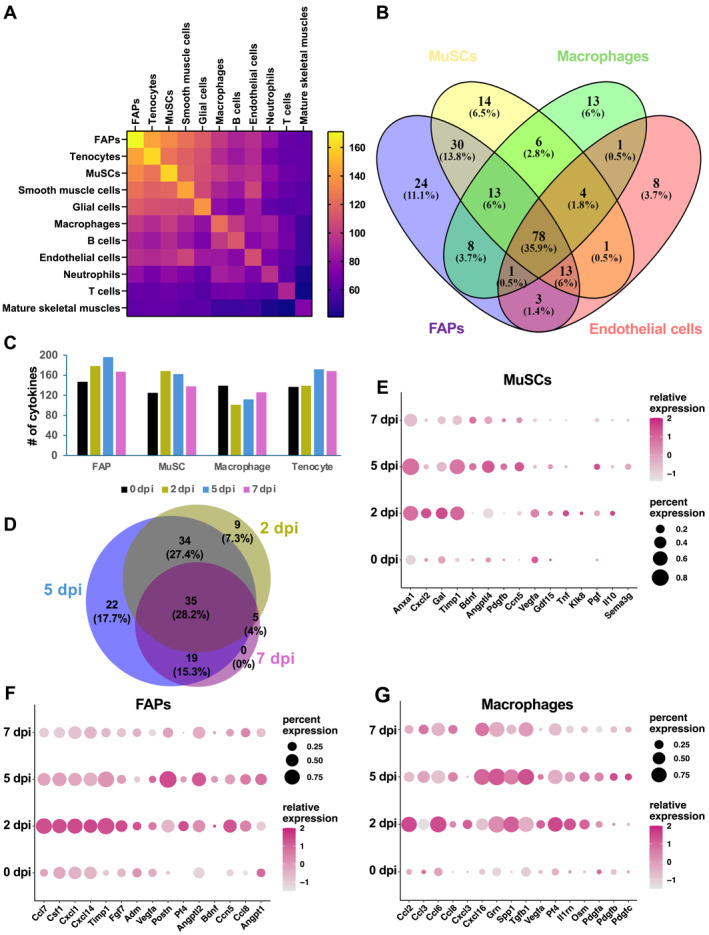
Regenerating muscles express a large array of cytokines. (A) A heatmap representing the number of cytokines detected in each cell type (diagonal axis) and number of overlapping cytokines between cell populations. Cell types are arranged in a descending order of number of cytokines detected. (B) A Venn diagram showing numbers of overlapping cytokines expressed by MuSCs, FAPs, macrophages, and endothelial cells. (C) Numbers of cytokines expressed in MuSCs, FAPs, macrophages, and tenocytes during regeneration. (D) A Venn diagram showing the numbers of upregulated cytokines in regenerating muscles (all cells combined) at 2, 5 and 7 dpi compared to 0 dpi. (E)–(G) Dot‐plots showing expression profiles of the most highly upregulated cytokines at 2, 5, and 7 dpi compared to 0 dpi in MuSCs (E), FAPs (F), and macrophages (G). Color gradient represents the average expression level and size of circle corresponds to percentage of cells in a cluster that expressed the gene.

To interrogate the integrated scRNA‐seq data for cytokine expression, we set a threshold of 1%, that is, a cytokine is considered to be expressed in a cell type if it is detected in at least 1% of the cells in the cluster. We examined the expression of 393 mouse cytokines (https://www.informatics. jax.org/) (Table S3) and found that 183 of them were expressed in our integrated datasets (Table S3). The highest numbers of cytokines were expressed in FAPs (171), tenocytes (161), MuSCs (160), smooth muscle cells (145), glial cells (138), and macrophages (124). We found a significant overlap of cytokines that were expressed in various cell types (Figure 2A). For instance, of the 171 cytokines expressed by FAPs, 134 were also expressed by MuSCs, whereas 101 cytokines overlapped between MuSCs and macrophages.

To determine whether any cytokines were exclusively expressed in one cell type, we compared cytokine expression among macrophages, FAPs, MuSCs, endothelial cells, and the cell types that contributed most to the integrated datasets. A Venn diagram showed that MuSCs expressed 14 unique cytokines (6.5% of MuSC‐expressed), whereas 24 (11.1%), 13 (6%), and 8 (3.7%) unique cytokines were observed in FAPs, macrophages, and endothelial cells, respectively (Figure 2B). Over the course of regeneration, the number of cytokines expressed in each cell type did not vary drastically (Figure 2C): FAPs and MuSCs expressed the highest numbers of cytokines at 2 dpi and 5 dpi, whereas macrophages expressed the most cytokines at 0 dpi; in tenocytes, higher numbers of cytokines were expressed at 5 dpi and 7 dpi.

To identify cytokines differentially expressed during regeneration, analysis was performed for 2, 5, and 7 dpi in comparison with 0 dpi. When compared to homeostatic muscles, 85, 112, and 61 cytokines were upregulated at 2, 5, and 7 dpi, respectively (Table S4). Among the upregulated cytokines, 35 appeared at all regenerating timepoints, whereas 9 and 22 cytokines were upregulated at 2 and 5 dpi, respectively (Figure 2D). We further examined differential cytokine expression in MuSCs, FAPs, and macrophages (Table S4); expression profiles of representative genes are shown in Figures 2E–2G. It is evident that within each cell type different genes were upregulated at different timepoints of regeneration. Similarly, dynamic patterns of cytokine expression throughout regeneration were also observed for other cell types (data not shown).

### 3.3 Extensive and dynamic cell‒cell communications in homeostatic and regenerating muscles

**FIGURE 3:**
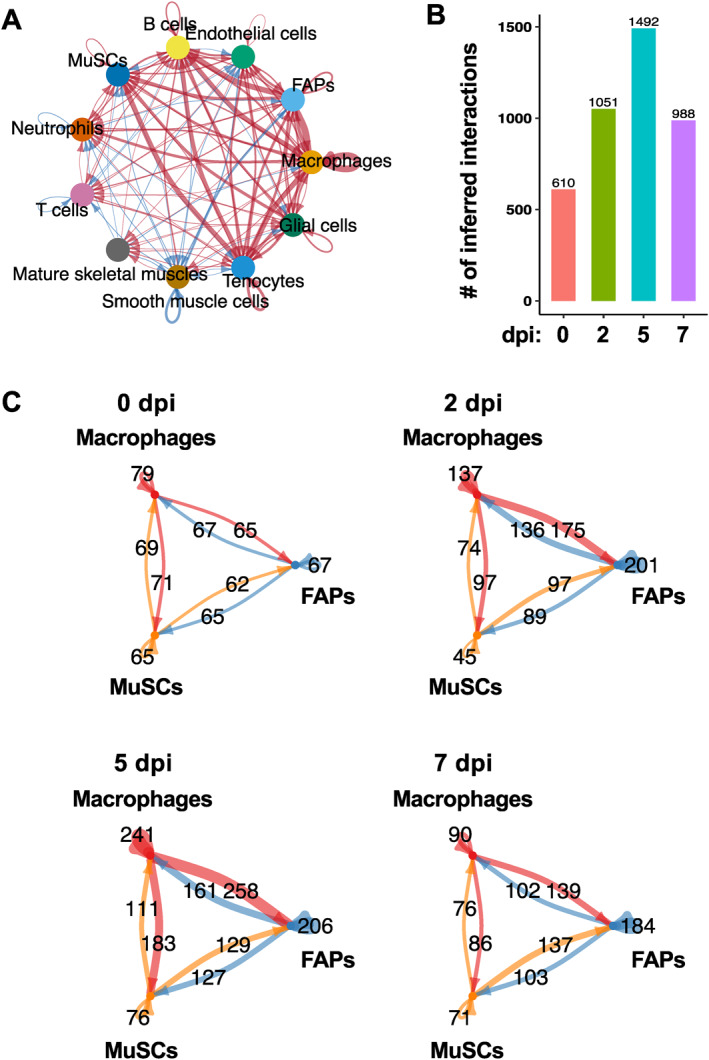
Cell–cell communication during skeletal muscle regeneration. (A) Chord‐diagram visualization of cell–cell communication networks involving all cell types identified in the integrated data. Arrow indicates direction of communication and line thickness corresponds to number of interactions. (B) Total numbers of inferred interactions at 0, 2, 5, and 7 dpi. (C) Numbers of communications between MuSCs, FAPs, and macrophages at 0, 2, 5, and 7 dpi.

To define the intercellular communication networks facilitated by secreted factors, we performed CellChat^28^ analysis, which included ∼1300 secreted factor‐mediated interactions (CellChatDB v2). We chose to use the stringent trimean method to identify only strong and highly significant interactions. Our analysis revealed a total of 4141 distinct cell–cell interactions that were predicted to be mediated by secreted factors during regeneration (Figure 3A). The total number of cytokine‐mediated interactions were highest at 5 dpi (1492), whereas the lowest number of interactions (610) were observed at 0 dpi (Figure 3B). We further zoomed in on the interactions among MuSCs, FAPs, and macrophages (Figure 3C). Predicted interactions between macrophages and FAPs increased more than two‐fold at 2 dpi from 0 dpi and further increased at 5 dpi. Indeed, 5 dpi saw the largest number of interactions involving macrophages with either FAPs or MuSCs. This number decreased drastically at 7 dpi, consistent with the idea that infiltrating macrophages mostly subsided at this stage of regeneration. The total number of interactions involving MuSCs (with both macrophages and FAPs) was also the highest at 5 dpi. The interactions were bi‐directional at every timepoint; there were substantial numbers of both macrophage–MuSC interactions and MuSC–macrophage interactions, and similarly for macrophage–FAP and FAP–MuSC interactions (Figure 3C, arrows indicating signaling direction).

**FIGURE 4:**
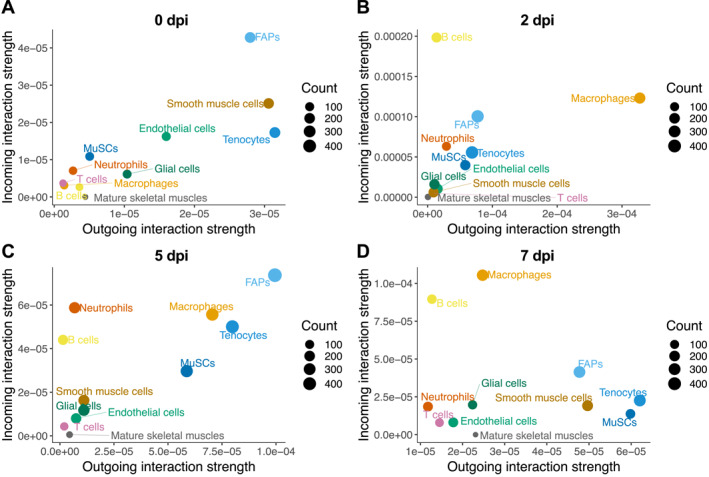
Contribution of cell types to sending and receiving cytokine signals during regeneration. (A)–(D) Scatter plots show signaling strength of various cell types in sending (outgoing, *x*‐axis) or receiving (incoming, *y*‐axis) cytokine signals at 0 dpi (A), 2 dpi (B), 5 dpi (C), and 7 dpi (D). Size of the circle represents total number of interactions (combining outgoing and incoming).

In addition to the number of interactions described above, we also used CellChat to analyze the interaction strength. Based on this parameter, FAPs were the major contributors to both sending signals (expressing cytokine) and receiving signals (expressing receptor) in homeostatic muscles (0 dpi, Figure 4A), whereas smooth muscle cells and tenocytes were also major signal senders. At 2 dpi (Figure 4B) macrophages were the major outgoing source of signals, whereas B cells received the most signals. At 5 dpi (Figure 4C), FAPs again became the major signal senders as well as receivers, followed by macrophages and tenocytes, whereas neutrophils and B cells were also major signal receivers. MuSCs also markedly increased their contributions to both sending and receiving signals at 5 dpi. Interestingly, at 7 dpi (Figure 4D) macrophages and B cells received the most signals, whereas tenocytes and MuSCs sent the most signals.

### 3.4 Cell type‐specific cytokine signaling before and during muscle regeneration

**FIGURE 5:**
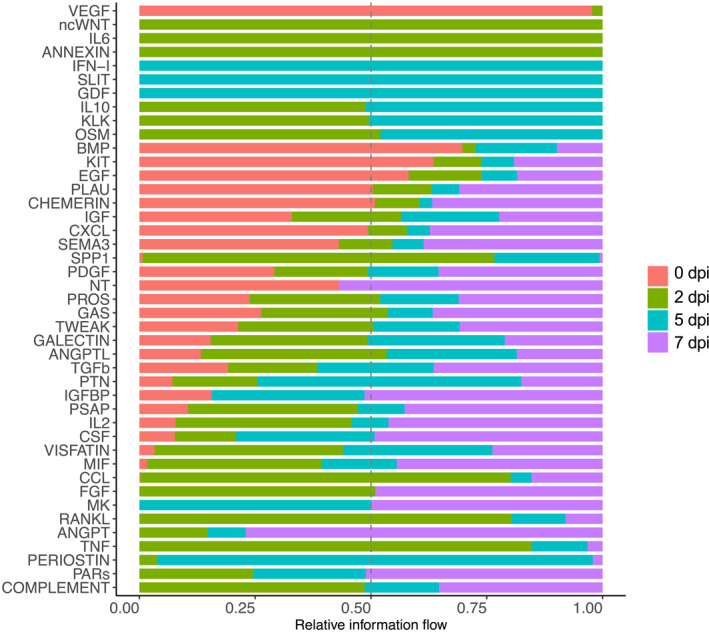
Overall information flow during regeneration. A paired Wilcoxon test was performed to identify signaling pathways with significant change over the course of regeneration. Relative strength of information flow is shown for 0, 2, 5, and 7 dpi.

To assess changes in specific signaling pathways over the course of regeneration, we examined information flow, which is defined as the sum of communication probabilities among all pairs of cell groups in the inferred networks.^28^ Pathways that underwent any significant change during regeneration were included in the analysis. As shown in Figure 5, signaling by some cytokines appeared at 2 dpi only (ncWNT, IL6, and ANNEXIN), 5 dpi only (IFN‐1, SLIT, and GDF), or both 2 and 5 dpi (IL10, KLK, and OSM). Some pathways were present at all the regeneration timepoints (2, 5, and 7 dpi) but not at 0 dpi, including CCL, FGF, MK, RANKL, ANGP, and PARs. VEGF signaling was unique, as the only pathway appearing exclusively in homeostatic muscles.

**FIGURE 6:**
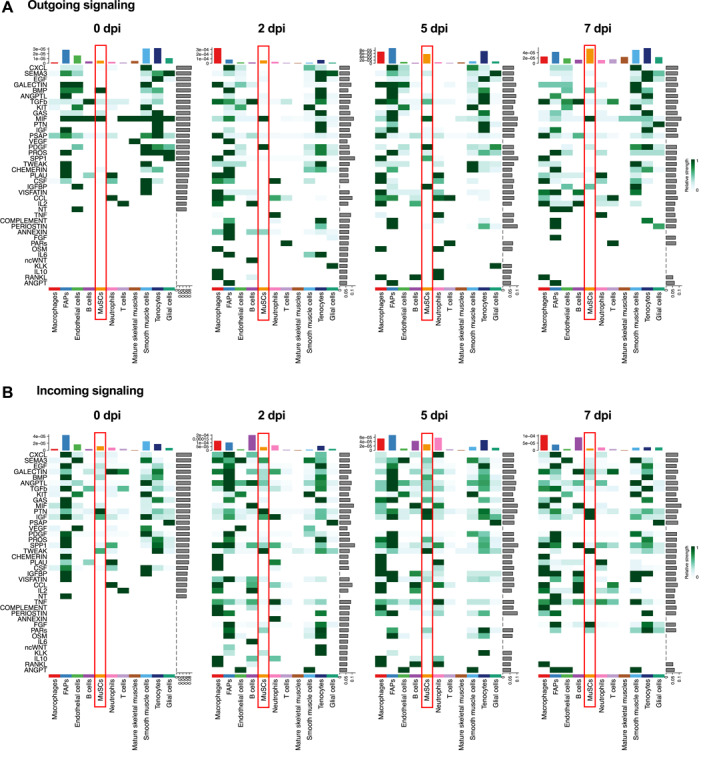
Outgoing and incoming signaling in individual cell types during regeneration. (A)–(B) Heatmap graphs show the outgoing (A) and incoming (B) signaling patterns at 0, 2, 5, and 7 dpi. Green gradient bars denote relative signaling strengths. Colored bars at the top represent the overall signaling strength of a particular cell type from summing all signaling pathways in the heatmap. Gray bars at the right of each graph represent the total signaling strength of a pathway from all cell types. MuSC data are highlighted by red boxes.

We then performed analysis of the outgoing (expressing cytokine) and incoming (expressing receptor) signaling by each of the 11 subpopulations of cells in homeostatic (0 dpi) and regenerating muscles (2, 5 and 7 dpi). As shown in Figure 6, we found some signaling to be constitutive and others to display temporal dynamics throughout regeneration. Take MuSC for example (see red boxes in Figure 6). Of the outgoing signals (Figure 6A), MIF signaling was present at all timepoints, including 0 dpi, whereas BMP signaling was restricted mostly to homeostatic muscles. Strong PDGF signaling from MuSCs appeared at 2 dpi and it was mostly gone by 7 dpi. Several other outgoing pathways were present in MuSCs at 5 dpi, including TGFβ, SPP1, and IGFBP. Of the signals that MuSCs were predicted to receive (Figure 6B), PTN stood out as a constitutive signal at all timepoints (0, 2, 5, and 7 dpi). Incoming TWEAK signaling was strong at all timepoints of regeneration but absent at 0 dpi. IGF signaling was also received by MuSCs at all timepoints, including 0 dpi, but the signaling strength was further increased at 5 and 7 dpi. Interestingly, incoming FGF signal was present exclusively at 7 dpi.

### 3.5 Communication between MuSCs and other cell types via PDGF, TGFβ, IGF, and TWEAK signaling

**FIGURE 7:**
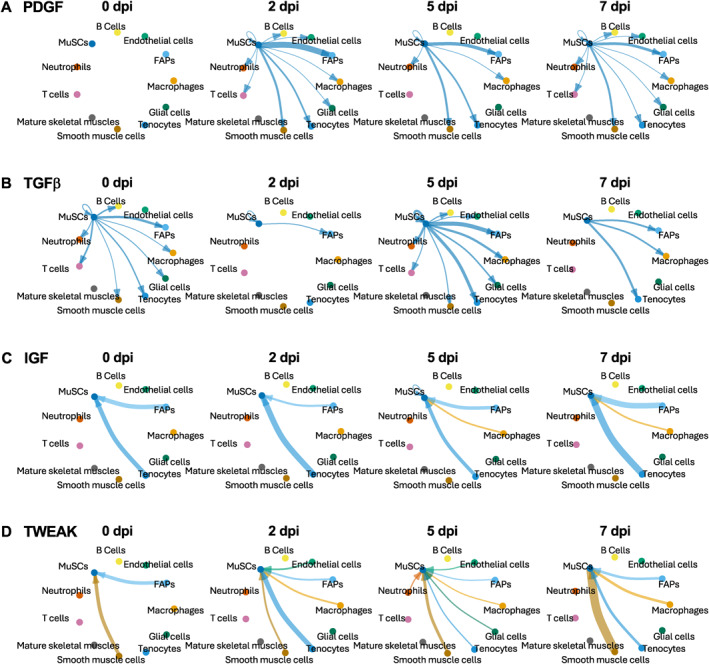
Examples of signaling to and from MuSCs over the early course of regeneration. (A)–(B) PDGF (A) and TGFβ (B) signaling from MuSCs to other cell types at 0, 2, 5, and 7 dpi. (C)–(D) IGF (C) and TWEAK (D) signaling from other cell types to MuSCs at 0, 2, 5, and 7 dpi. Thickness of the lines corresponds to relative signaling strength and arrow indicates the direction of signaling.

Further expanding the analysis above to predict the cell populations that receive or send signals from or to MuSCs, we highlight four signaling pathways: PDGF, TGFβ, IGF, and TWEAK (Figure 7). Known to promote myoblast proliferation and inhibit myogenic differentiation in vitro,^17^ and to be expressed by MuSCs in vivo,^29^ the role of MuSC‐produced PDGF in muscle regeneration is unclear. Our analysis revealed that PDGF signaling from MuSCs occurred exclusively during regeneration and not in homeostatic muscles, and that FAPs were the major receivers of PDGF signal although several other cell types were also involved in the receiving (Figure 7A). Similar to PDGF, TGFβ also promotes myoblast proliferation and inhibits myogenic differentiation in vitro,^17^ as well as negatively regulates myocyte fusion in vivo.^30^ Moreover, similar to PDGF, what role MuSC‐sourced TGFβ plays in muscle regeneration is not known. We found prevalent TGFβ signaling from MuSCs to FAPs and tenocytes, as well as autocrine signaling, at 5 dpi (Figure 7B). Early activation of autocrine TGFβ signaling aligns with the notion of promoting MuSC proliferation and inhibiting differentiation, but TGFβ signaling from MuSCs to FAPs and tenocytes warrant future investigation.

Studies across diverse animal and cellular models indicate that IGFs play crucial roles in regulating skeletal muscle development as well as homeostasis, hypertrophy, and regeneration of adult muscle tissues.^31^, ^32^, ^33^ Despite the long‐standing notion of autocrine IGF signaling in muscle cells based on numerous in vitro studies,^17^ evidence for a muscle cell‐derived IGF function in vivo is still absent. Our analysis suggested that MuSCs predominantly served as a receiver rather than a sender of IGF signals, mostly from tenocytes and FAPs at all timepoints of regeneration and in homeostatic muscles, and also from macrophages at 5 and 7 dpi (Figure 7C). The TNF family member TWEAK has been reported to promote myoblast proliferation while inhibiting terminal differentiation in vitro.^34^ Expression of TWEAK increased upon muscle injury,^34^, ^35^ and it was suggested that this expression mostly came from macrophages.^34^ Systemic knockout of TWEAK in mice led to enhanced muscle regeneration,^35^ but mice deficient in Tn14, a receptor of TWEAK, displayed impaired muscle regeneration upon injury, presumably due to a perturbed inflammatory response.^34^ A cell type‐specific function of TWEAK in vivo is not reported. Surprisingly, our results indicated that TWEAK signaling to MuSCs came from smooth muscle cells at all timepoints with the highest strength at 7 dpi, as well as from FAPs at 0 dpi and from tenocytes at 2 dpi (Figure 7D). These predictions of informational flow can guide future investigations to yield novel insights in the regulation of muscle regeneration.

### 3.6 Cytokine‐receptor signaling to MuSCs during muscle regeneration

**FIGURE 8:**
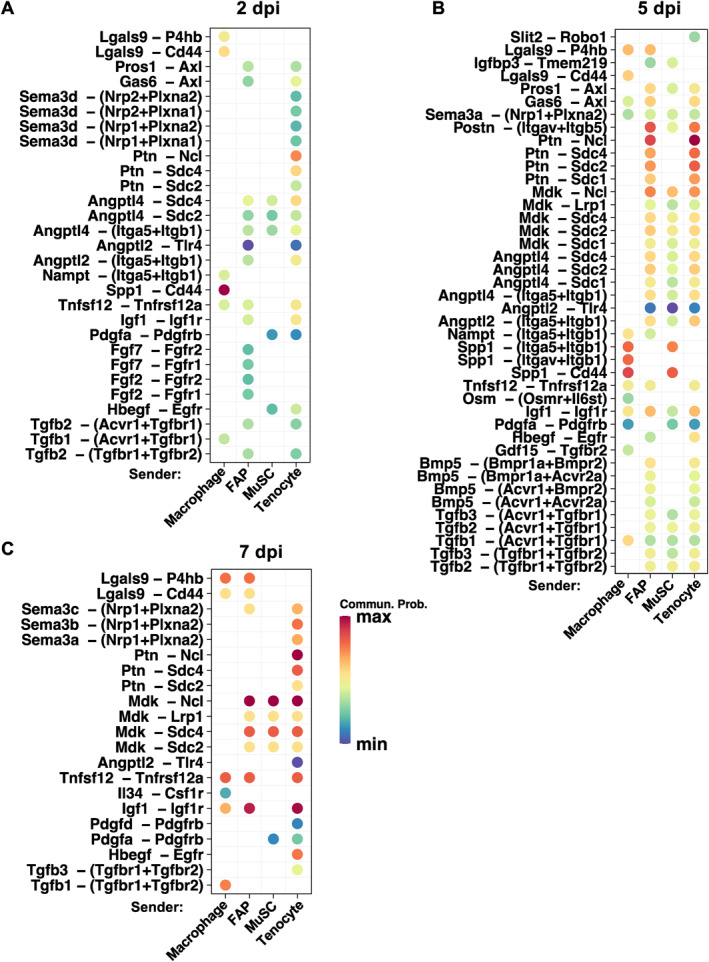
Cytokine signaling to MuSCs upregulated in regenerating muscles. (A)–(C) Upregulated ligand–receptor signaling to MuSCs from macrophages, FAPs, tenocytes, and MuSCs at 2 dpi (A), 5 dpi (B), and 7 dpi (C) compared to 0 dpi. Relative communication probability is represented by color scheme as indicated. *p*‐value threshold was set to <0.01 to identify significantly upregulated signaling.

Finally, we were interested in the signaling to MuSCs upregulated in regenerating muscles and the sources of the signaling, particularly from FAPs, macrophages, tenocytes, or MuSCs (autocrine). Ligand‒receptor interactions significantly upregulated at 2, 5, and 7 dpi from 0 dpi are shown in Figure 8. The number of upregulated ligand–receptor interactions with high probability was at the lowest at 2 dpi and highest at 5 dpi. Among the upregulated ligand‒receptor pairs at 2 dpi communication from macrophages to MuSCs mediated by Spp1‐Cd44 had the highest probability (Figure 8A), and this signaling remained at 5 dpi while also appeared as autocrine signaling in MuSCs (Figure 8B). Interestingly, even though overall Ptn signaling appeared to be constitutive (Figure 6B), specific Ptn‐receptor signaling received by MuSCs were upregulated during regeneration, especially Ptn‐Ncl signaling from tenocytes at all regeneration time points (Figure 8A–C) and also from FAPs at 5 dpi (Figure 8B). The large number of highly upregulated signaling to MuSCs at 5 dpi (Figure 8B) potentially offer new insights into the myogenic process in vivo. The significant reduction in the number of upregulated ligand‒receptor pairs at 7 dpi (Figure 8C) is not surprising, as new myofiber formation has reached full force by that time. It is plausible that the signaling pathways at 7 dpi are mainly engaged in myofiber maturation and/or clearance of immune cells.

## 4 DISCUSSION

The contribution of distinct types of cells to the cytokine milieu in the injured skeletal muscle essential for regulating regeneration and repair has been well recognized. An atlas of cytokine expression at the single‐cell level in the regenerating muscle would be invaluable for a comprehensive view of potential regulatory roles of cytokines and the cellular interplay that underlie successful regeneration. Interrogating scRNA‐seq datasets publicly available, we have performed deep analyses of cytokine expression in homeostatic and regenerating muscles. We find nearly one half of the ∼400 curated cytokines to be expressed in the skeletal muscle. Whereas a few cytokines are expressed exclusively in each cell type, most cytokines are expressed in multiple cell types, although the temporal dynamics of expression for many of those cytokines are cell type‐specific. When combined across the early stage of muscle regeneration from day 0 to day 7 post injury, FAPs express the highest number of distinct cytokines, closely followed by tenocytes and MuSCs (Figure 2A). It is remarkable that 160 distinct cytokines are found to be expressed by the MuSC, a cell type not traditionally associated with cytokine secretion. Because scRNA‐seq is prone to low coverage and the level of cytokine expression in MuSC is also typically low, it is reasonable to assume that the actual number of MuSC‐expressed cytokines is even larger.

Analysis of the expression of receptors in addition to cytokines reveals potential cell–cell communications over the course of muscle regeneration, suggesting an intricate network of cytokine signaling that may govern the sequential events initiated by muscle injury and ending in the formation of new myofibers. Not surprisingly, the number of predicted cell–cell interactions is higher in regenerating muscles than in homeostatic muscles, and highest at 5 dpi coinciding with regulation of MuSC differentiation and a transition from pro‐inflammatory to anti‐inflammatory microenvironment.^5^, ^10^, ^36^ The largest number of predicted interactions at 5 dpi are from macrophages to MuSCs and FAPs, but the numbers of interactions initiated from MuSCs and FAPs are also substantial. The bi‐directionality of interactions among these major cell types is interesting, and the emerging concept of MuSCs as immunomodulators during muscle regeneration ^37^ is supported by the numerous communications predicted to occur from MuSCs to macrophages. Future investigation of the individual cytokine–receptor pairs can potentially shed light on how MuSCs exert immunomodulatory functions. It is also noteworthy that a subset of MuSCs enriched in immune gene expression, named immunomyoblasts, has been identified by Oprescu et al. ^18^ and Yartseva et al.^38^ Future analysis of the integrated scRNA‐seq data for cytokine expression and cell–cell interaction by immunomyoblasts should prove illuminating.

One of the strong interactions involving MuSC is IGF, and it is present during homeostasis as well as at every time point of early regeneration (Figure 7C). The critical function of IGF1 and IGF2 in muscle development, hypertrophy, and regeneration has been well established,^39^, ^40^, ^41^ and IGFs are among the earliest reported autocrine factors that regulate myogenic differentiation.^42^, ^43^, ^44^ However, the autocrine function of IGF has never been experimentally proven in vivo. In our analysis, MuSC is predicted to almost exclusively receive IGF signals from FAPs and tenocytes, with a low degree of autocrine signaling at 5 dpi and also some signaling coming from macrophages at 5 and 7 dpi (Figure 7C). Hence, it appears that an autocrine function of IGF in myogenesis in vivo that mirrors the well‐reported autocrine IGF signaling in vitro is unlikely. However, the high stringency of CellChat prediction in our analysis means that only the strongest interactions are revealed. It is likely that our study has missed weaker but nonetheless biologically meaningful interactions, especially those involving MuSC‐derived cytokines, which are typically expressed at lower levels. Only MuSC‐specific depletion of IGF can definitively prove or rule out an autocrine function in vivo. It is also worth noting that a lot more paracrine signaling events are predicted than autocrine signaling for MuSC‐derived cytokines (Figure 3C), which calls into question the effectiveness or reliability of the popular approach of studying muscle cell‐derived cytokines in vitro where only autocrine functions are assessed.

## 5 CONCLUSION

Mining publicly available scRNA‐seq datasets, our study provides novel insights into cytokine expression by various types of cells in the injury‐induced regenerating muscle niche. We find large numbers of distinct cytokines expressed by almost all types of cells including MuSCs, FAPs, immune cells, tenocytes, and endothelial cells. Our analysis also predicts extensive and dynamic cell–cell communications via cytokine signaling during the early phase of muscle regeneration, some previously reported but many not yet explored experimentally. Our study demonstrates the power of interrogating existing transcriptomic datasets. The outcome of this study and future analysis of ever‐expanding datasets will provide important resources to guide experimental studies that can lead to a deep understanding of cytokine signaling network in muscle regeneration.

## AUTHOR CONTRIBUTION

**PB** and **JC** conceptualized the study; **PB** performed data analysis and made figures; **PB** and **JC** interpreted the data and wrote the manuscript; both authors revised the manuscript and approved the final version.

## CONFLICT OF INTEREST STATEMENT

The authors declare that they have no conflict of interest.

## ETHICS STATEMENT

This study utilized only publicly available data and did not involve any animals or human subjects.

## Supporting information

Supplementary Material S1

Supplementary Table S1

Supplementary Table S2

Supplementary Table S3

Supplementary Table S4

## Data Availability

Data sharing is not applicable to this article as no new data were created or analyzed in this study.
