# Supplementary material for: Cytokine expression and cytokine‐mediated cell–cell communication during skeletal muscle regeneration revealed by integrative analysis of single‐cell RNA sequencing data
Source: J Cell Commun Signal. 2024 Dec 4;18(4):e12055. doi: 10.1002/ccs3.12055 (PMC11647049; doi:10.1002/ccs3.12055)
Supplement: Supplementary file 1 — Supplementary Material S1 [file CCS3-18-e12055-s003.pdf]

SUPPLEMENTARY TABLES

Table S1. Marker genes for identification of the 11 cell types in regenerating muscles.

Table S2. Source of datasets analyzed in this study.

Table S3. Cytokine genes analyzed in this study. (1) A complete list of 393 cytokines analyzed in the study (https://www.informatics.jax.org/). (2) Percentage of cells expressing each cytokine (1% is used as the cutoff for expression).

Table S4. Cytokine genes differentially expressed in regenerating muscles. (1) Cytokines differentially expressed at 2, 5, or 7 dpi compared to 0 dpi, combing all cell types. (2) Cytokines differentially expressed in MuSCs at 2, 5, or 7 dpi compared to 0 dpi. (3) Cytokines differentially expressed in macrophages at 2, 5, or 7 dpi compared to 0 dpi. (4) Cytokines differentially expressed in FAPs at 2, 5, or 7 dpi compared to 0 dpi.

SUPPLEMENTARY FIGURES

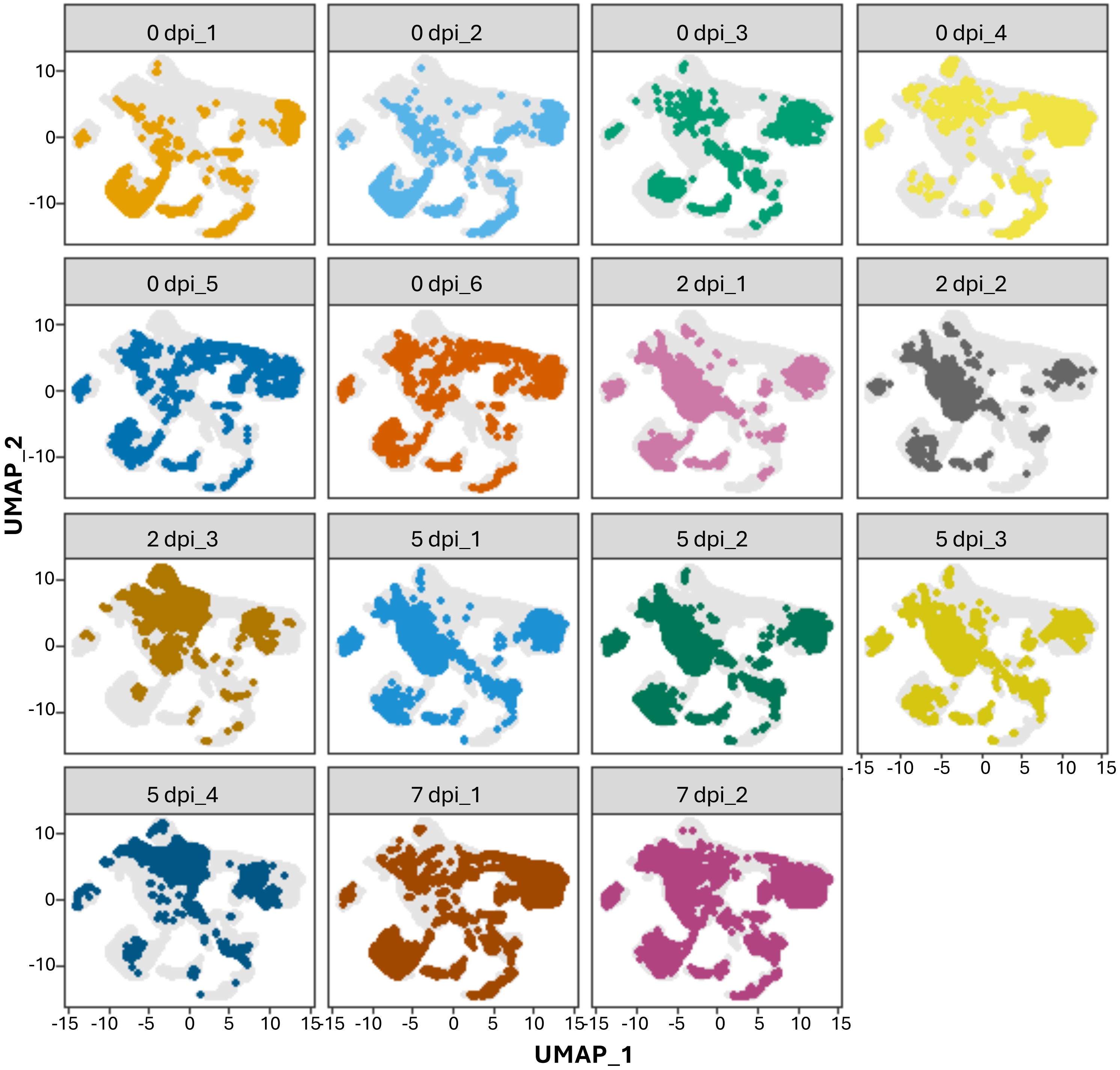

Figure S1. UMAP visualization of individual scRNA-seq datasets before integration. Datasets were visualized by UMAP plotting before integration to identify potential batch effects.

A

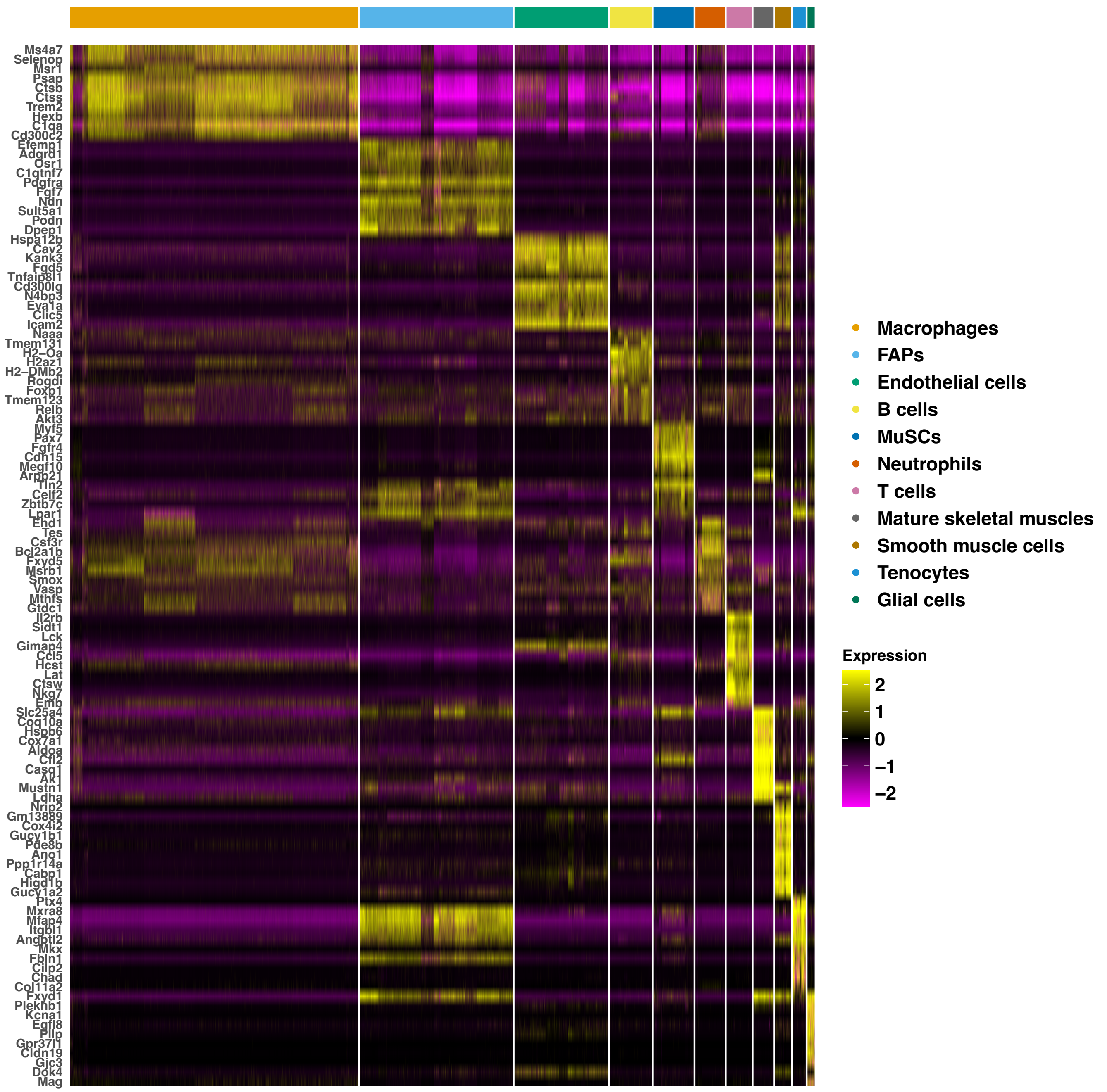

B

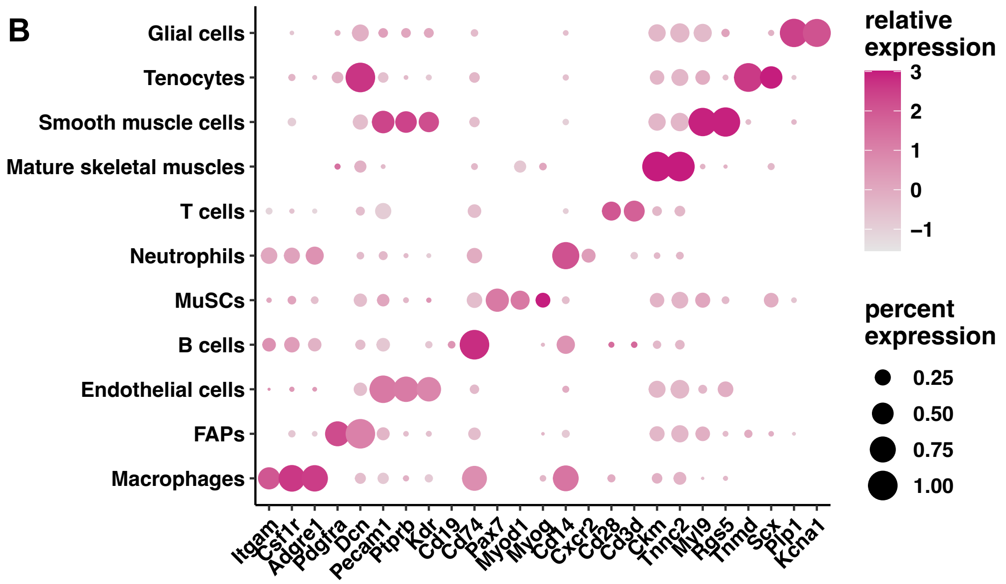

Figure S2. Characterization of cell clusters in the integrated dataset. **(A)** Top 10 enriched genes for each cluster are shown in the heatmap. **(B)** Expression patterns of marker genes for various cell clusters are shown in the dot-plot. Color gradient represents the average expression level and size of circle corresponds to percentage of cell cluster expressing the gene.

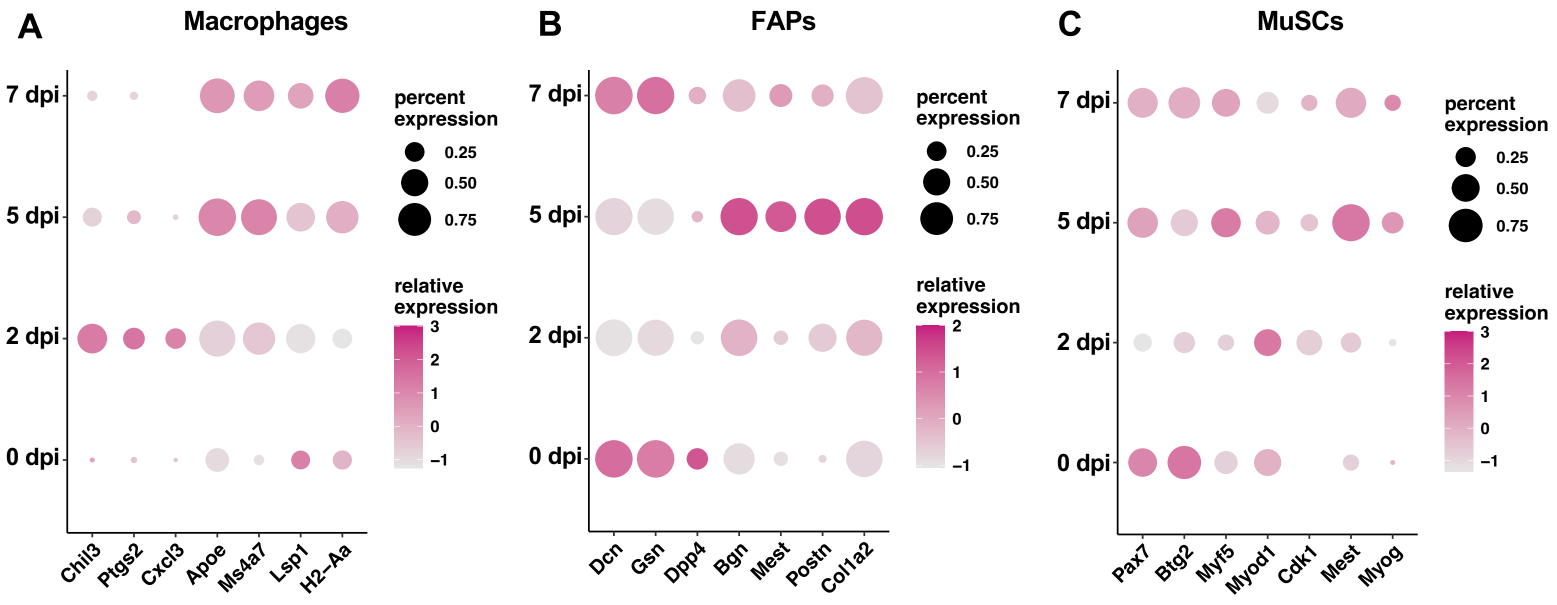

Figure S3. Gene expression dynamics during regeneration. **(A-C)** Dot-plots show the expression of various injury responsive genes in macrophages (A), FAPs (B) and MuSCs (C) at 0, 2, 5, and 7 dpi. Color gradient represents the average expression level and size of circle corresponds to percentage of cell cluster expressing the gene.
